# A new species of *Hemicorallium* (Anthozoa, Octocorallia, Coralliidae), along with a revised identification key to species of the genus

**DOI:** 10.3897/zookeys.1277.184620

**Published:** 2026-04-20

**Authors:** Tatsuki Koido

**Affiliations:** 1 Kuroshio Biological Research Foundation, 560 Nishidomari, Otsuki, Kochi 788-0333, Japan Kuroshio Biological Research Foundation Otsuki Japan

**Keywords:** Cnidaria, deep-sea corals, precious coral, Scleralcyonacea, Western Pacific

## Abstract

A new precious coral species, *Hemicorallium osmanthogemmum***sp. nov**. (Scleralcyonacea, Coralliidae), retrieved from a depth of 550 m in Kochi Prefecture, Japan, is described, and an updated identification key for the genus *Hemicorallium* is provided. The new species is confirmed as belonging to the genus *Hemicorallium* based on the presence of nonretractile but contractile autozooids with an ovate-cylindrical shape and sclerites comprising elongated rods, 6–8-radiates and double clubs, with elongated rods present exclusively in the tentacles. Moreover, the species is distinct from all its congeners in that it has orange coenenchyme and a pink axis, with autozooids distributed only on the front side of the branch, forming clusters at the branch tips. These morphological features differ from those of all previously described species of *Hemicorallium*, and molecular analysis of mitochondrial sequences (16S, ND1, ND2, ND3, ND6, COI, MSH, and IGR1) further corroborates the validity of this new species. This is the first report of *Hemicorallium* off the coast of Kochi Prefecture, Japan.

## Introduction

Precious corals, primarily belonging to the family Coralliidae in the subclass Octocorallia, have long been valued for their beautiful axial skeletons and vivid colouration, which make them suitable for jewellery and ornaments. In recent years, efforts have shifted not only toward resource consumption, but also toward developing methods for propagation (Weinberg 1979; Montero-Serra et al. 2018; Boch et al. 2019; Koido et al. 2022). Moreover, in Japan, precious corals had already attracted scientific attention in the early 20th century (Kishinouye 1903a, 1904), and resource conservation through coral release was considered over a century ago (Nagamune 1918; Kuno 1922), reflecting a longstanding interest in the sustainable development of the precious coral industry.

The family Coralliidae was classified into three genera by Tu et al. (2015b) based on morphological and molecular phylogenetic data; however, a more recent study by McFadden et al. (2022) restructured the group to comprise 14 genera by integrating 11 molecularly supported genera. Furthermore, two new genera—*Mollecorallium* Altuna & López-González, 2023 and *Neoanthomastus* Li et al., 2025—have been described, resulting in a total of 16 genera. These changes indicate that the taxonomy of Coralliidae is currently undergoing major revision. The genus *Hemicorallium* Gray, 1867 includes species that inhabit particularly deep waters, and sampling opportunities are limited. Therefore, many undescribed species in this group are thought to exist. In the present study, we describe a previously unknown species of precious coral collected off Cape Ashizuri, Kochi Prefecture, Japan, based on detailed morphological observations and molecular phylogenetic analyses. Clarifying the taxonomic placement of such unknown precious corals will contribute to a more accurate assessment of their natural populations and support better management and conservation strategies.

## Materials and methods

### Sample origin

Two colonies were collected off Cape Ashizuri, Kochi Prefecture, Japan (to protect the collection site, the precise location data are not disclosed), after being incidentally hooked during alfonsino (*Beryx splendens* Lowe, 1834) fishing operations at a depth of approximately 550 m. The specimens were deposited in a dried state at the Kuroshio Biological Foundation (KBF) in Kochi and registered in the Octocoral Collection (OA) under the accession numbers KBF-OA-00436 and KBF-OA-00437. Coenenchymal tissue samples from both specimens were preserved in 99% ethanol for molecular phylogenetic analysis.

### Morphological observation

Morphological observations were conducted to examine the following: the arrangement, height, and diameter of the polyps; the presence or absence of polyp clusters; the surface structure of the coenenchyme and axial skeleton; and the presence of polychaete borings. These features were examined using a stereo microscope (SZX16, Olympus Corp.) and a fluorescence microscope (BX50, Olympus Corp.). Sclerites were extracted from different regions of the colony, and their shapes, sizes, and regionally dominant types were examined using a scanning electron microscope (SEM; JSM-6500F, JEOL Ltd).

### DNA extraction, amplification, and sequencing

DNA was extracted from ethanol-preserved tissue samples using a DNeasy Blood & Tissue Kit (Qiagen). Eight genetic markers were used that were demonstrated to be effective in distinguishing precious coral species: 16S, ND1, ND2, ND3, ND6, COI, MSH, and IGR1 (Tu et al. 2015a, 2015b; Nonaka and Hayashibara 2021; Nonaka et al. 2023). Primer sequences for each marker are listed in Table 1. In addition to the six markers used by Tu et al. (2015b) and Nonaka et al. (2023), *Paragorgia* Milne Edwards, 1857, a genus closely related to Coralliidae, was included as the outgroup (Table 2). Phylogenetic trees were constructed using maximum likelihood (ML) analysis in MEGA11 (Tamura et al. 2021) and Bayesian inference (BI) in MrBayes software v. 3.2.1 (Ronquist et al. 2012). For ML analysis, the T92+I model was selected as the best-fit substitution model, and 1,000 bootstrap replicates were used to assess nodal support. For BI analysis, the concatenated alignment data were treated as separate data partitions. The analysis was run for 10 million generations, with a burn-in of 25%, using default Metropolis-coupled Markov chain Monte Carlo parameters, until the standard deviation of split frequencies fell below 0.01.

**Table 1.** Primer sequences applied to Coralliidae samples, with corresponding references.

Region	Primer	Sequence (5'–3')	Source
16S rRNA	Octo1-L	AGACCCTATCGAGCTTTACTGG	France et al. (1996)
	Octo2-H	CGATAAGAACTCTCCGACAATA	France et al. (1996)
ND1	ND1-Co-F	AAGAGATGGCTATTCCCAAT	Tu et al. (2015b)
	ND1-Co-R	ATCAACAGGCCAGAAACTAA	Tu et al. (2015b)
16S rRNA–ND2	16S544F	CGACCTCGATGTTGAGTTGCGG	McFadden et al. (2006)
	ND21418R	ACATCGGGAGCCCACATA	McFadden et al. (2004)
ND3–ND6	ND61487F	TTTGGTTAGTTATTGCCTTT	McFadden et al. (2004)
	ND32126R	CACATTCATAGACCGACACTT	McFadden et al. (2004)
ND6–COI	IGR1-Co-F	AAACAGGATTCGGGGTAGA	Tu et al. (2015b)
	IGR1-Co-R	GCCATTCCGGAAAATGCT	Tu et al. (2015b)
MSH	MSH-Co-F	ACAGGGGAATGCTAATACTCTCAG	Tu et al. (2015b)
	MSH-Co-R	TAGAAGGACGGAATACTGGTAAGG	Tu et al. (2015b)

**Table 2.** List of specimens of the family Coralliidae examined in this study and accession numbers for 16S rRNA, 16S rRNA–ND2, partial ND1, partial MSH, ND3–ND6, and IGR1 markers.

Specimen ID	Voucher ID	16S	ND1	16S–ND2	ND3–ND6	ND6–COI	MSH	Reference
*Hemicorallium osmanthogemmum*	KBF-OA-00436	LC910899	LC910900	LC910901	LC910902	LC910903	LC910904	This study
*Hemicorallium osmanthogemmum*	KBF-OA-00437	LC910905	LC910906	LC910907	LC910908	LC910909	LC910910	This study
*Corallium japonicum*	ASIZ 80303	KF850198	KF854776	KF850283	KF850341	KF855041	KF854882	Tu et al. (2015b)
*Corallium nix*	USNM 1153916	KF417750	KF854833	KF417751	KF417752	KF855010	KF854939	Tu et al. (2015b)
*Corallium rubrum*	ASIZ 80406	KF286554	KF854765	KF286555	KF286556	KF855039	KF854871	Tu et al. (2015a, 2015b)
*Corallium tortuosum*	MNHN-IK-2011-1462	KF850173	KF854814	KF850291	KF850358	KF855011	KF854920	Tu et al. (2015b)
*Hemicorallium abyssale* (1)	USNM 1072424	N.A.	KF854815	N.A.	N.A.	KF854996	KF854921	Tu et al. (2015b)
*Hemicorallium abyssale* (2)	USNM 1164629	KF850208	KF854763	KF850279	KF850335	KF854981	KF854869	Tu et al. (2015b)
*Hemicorallium aurantiacum*	USNM 11952201	KF850175	KF854812	KF850295	KF850357	KF854995	KF854918	Tu et al. (2015b)
*Hemicorallium bathyrubrum*	USNM 1135989	KF850178	KF854808	KF850292	KF850308	KF854992	KF854914	Tu et al. (2015b)
*Hemicorallium bayeri*	USNM 1090559	N.A.	KF854780	N.A.	N.A.	KF854964	KF854886	Tu et al. (2015b)
*Hemicorallium ducale* (1)	USNM 94457	KF850232	KF854764	KF850280	KF850336	KF855025	KF854870	Tu et al. (2015b)
*Hemicorallium ducale* (2)	MNHN-IK-2011-1709	KF850213	KF854754	KF850274	KF850330	KF854978	KF854860	Tu et al. (2015b)
*Hemicorallium guttatum* (1)	USNM 1175422	KF850211	KF854756	KF850276	KF850312	KF855006	KF854862	Tu et al. (2015b)
*Hemicorallium guttatum* (2)	USNM 1116818	N.A.	KF854773	N.A.	N.A.	KF855007	KF854879	Tu et al. (2015b)
*Hemicorallium imperiale* (1)	SNM 1116815	N.A.	KF854737	N.A.	N.A.	KF855020	KF854843	Tu et al. (2015b)
*Hemicorallium imperiale* (2)	USNM 98780	KF850169	KF854825	KF850297	KF850361	KF854975	KF854931	Tu et al. (2015b)
*Hemicorallium indicodensum*	DY72JL22003	PQ096168	PQ106789	N.A.	N.A.	PQ106791	PQ106787	Hu et al. (2025)
*Hemicorallium jiaolongensis*	DY72JL22005	PQ096169	PQ106790	N.A.	N.A.	PQ106792	PQ106788	Hu et al. (2025)
*Hemicorallium laauense*	USNM 1072446	KF850210	KF854760	KF850277	KF850332	KF855024	KF854866	Tu et al. (2015b)
*Hemicorallium niobe*	USNM 1110365	N.A.	KF854799	N.A.	N.A.	KF854965	KF854905	Tu et al. (2015b)
*Hemicorallium regale*	USNM 1114527	N.A.	KF854826	N.A.	N.A.	KF855000	KF854932	Tu et al. (2015b)
*Hemicorallium sulcatum*	NMNS 6606002	KF850191	KF854795	KF850269	KF850349	KF854988	KF854901	Tu et al. (2015b)
*Hemicorallium meraboshi*	NSMT:Co:1801	LC716760	LC716762	LC716763	LC716764	LC770918	LC716761	Nonaka et al. (2023)
*Pleurocorallium bonsaiarborum*	USNM 1195214	KF850181	KF854805	KF850267	KF850353	KF854957	KF854911	Tu et al. (2015b)
*Pleurocorallium borneense*	MNHN-IK-2011-1542	KF850218	KF854750	KF850236	KF850326	KF854973	KF854856	Tu et al. (2015b)
*Pleurocorallium carusrubrum*	ASIZ 0000960	KF483567	KF854802	KF483568	KF483569	KF855038	KF854908	Tu et al. (2015b)
*Pleurocorallium clavatum*	USNM 1195223	KF850183	KF854803	KF850240	KF850351	KF855003	KF854909	Tu et al. (2015b)
*Pleurocorallium elatius*	ASIZ 80411	KF850201	KF854772	KF850258	KF850301	KF855028	KF854878	Tu et al. (2015b)
*Pleurocorallium inutile*	ASIZ 80317	KF286557	KF854792	KF286558	KF286559	KF854943	KF854898	Tu et al. (2015b)
*Pleurocorallium konojoi*	ASIZ 80340	KF850184	KF854800	KF850266	KF850307	KF855032	KF854906	Tu et al. (2015b)
*Pleurocorallium niveum*	USNM 56807	KF850192	KF854786	KF850239	KF850346	KF854952	KF854893	Tu et al. (2015b)
*Pleurocorallium norfolkicum*	MNHN-IK-2011-1474	KF850222	KF854747	KF850250	KF850323	KF854949	KF854853	Tu et al. (2015b)
*Pleurocorallium porcellanum*	USNM 1164644	KF850174	KF854813	KF850242	KF850309	KF855017	KF854919	Tu et al. (2015b)
*Pleurocorallium secundum*	ASIZ 80388	KF850194	KF854784	KF850238	KF850310	KF854951	KF854890	Tu et al. (2015b)
*Pleurocorallium thrinax*	MNHN-IK-2011-1587	KF850227	KF854739	KF850246	KF850315	KF854959	KF854845	Tu et al. (2015b)
*Paragorgia* sp.	USNM 1075769	KC782349	KC782349	KC782349	N.A.	KC782349	KC782349	Figueroa and Baco (2014)
*Paragorgia* sp.	USNM 1075761	KC782350	KC782350	KC782350	N.A.	KC782350	KC782350	Figueroa and Baco (2014)

## Results

### Systematics

**Class Octocorallia Haeckel, 1866**

**Order Scleralcyonacea McFadden, van Ofwegen & Quattrini, 2022**

**Family Coralliidae Lamouroux, 1812**

#### Genus *Hemicorallium* Gray, 1867

##### Hemicorallium osmanthogemmum sp. nov.

Taxon classificationAnimaliaScleralcyonaceaCoralliidae

1493EC49-2A43-534E-A930-E0045E4EF9C0

https://zoobank.org/DE9B02CB-8A7D-456C-80DD-ACD8A0BEB632

Figs 1, 2, 4, 5

###### New Japanese name.

Kinmokusei-mizo-sango.

###### Type material.

***Holotype***: KBF-OA-00436; Off Cape Ashizuri, Tosashimizu City, Kochi Prefecture, depth 550 m, July 2016, coll. Keiji Takami. ***Paratype***: KBF-OA-00437, Off Cape Ashizuri, Tosashimizu City, Kochi Prefecture, depth 550 m, July 2016, coll. Keiji Takami.

###### Diagnosis.

The colony branches in one plane, and anastomoses are present only where broken branches come into contact with other branches. Autozooids are contracted but not retracted, and they are distributed only on one side of the colony. Spacing is almost absent on the twigs, and clusters are formed at the tips. The axis has no pits beneath the autozooids and is pale pink to pink in colour. Sclerites are predominantly radiate, and double clubs are rare. Warty rods are present only in the tentacles.

###### Description of holotype.

**Figure 1.**
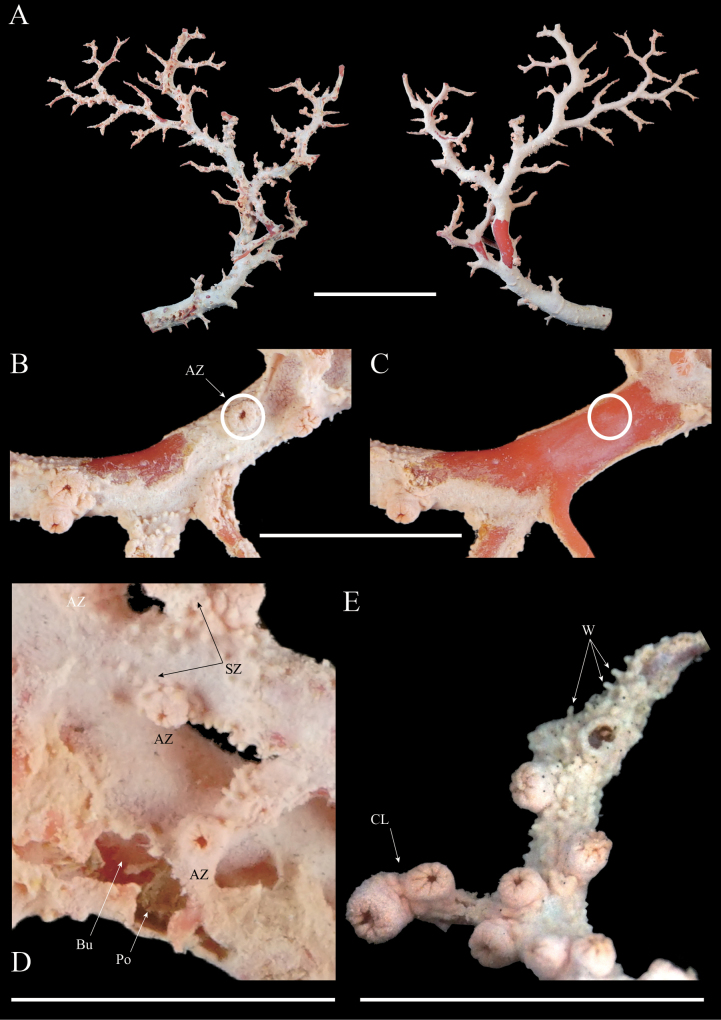
*Hemicorallium osmanthogemmum* sp. nov. (Holotype: KBF-OA-00436). **A**. ‘‘Front’’ and ‘‘back’’ of colony; **B**. Branch, showing autozooid (AZ); **C**. Exposed axis with the coenenchyme removed; **D, E**. Surface detail of branch, showing autozooid (AZ), siphonozooid (SZ), burrows (Bu), cluster of autozooids (CL), warts on coenenchyme surface (W), and commensal polychaete (Po). Scale bars: 50 mm (**A**); 10 mm (**B–E**).

***Colony***. The holotype colony is fan-shaped and preserved in a dried state. It is approximately 12 cm in height and 13 cm in width. The colony branches in one plane from the main stem; partial fusion is present at sites of branch fracturing, and direct fusion between intact branches is generally absent (Fig. 1A). The basal diameter is 9.3 mm, and the cross-section is nearly circular. The surface of the coenenchyme is covered with conical protuberances that become more pronounced toward the branch tips, forming longitudinal grooves as the projections align (Fig. 1E).

***Polyps***. Contracted autozooids are restricted to one side of the colony (Fig. 1A) and occasionally form clusters of two or three polyps at the branch tips (Fig. 1E). On the stem, autozooids are typically spaced approximately 4–5 mm apart; however, some occur in direct contact with each other, whereas others are separated by more than 10 mm. They do not completely retract into the coenenchyme, but contracted autozooids form cylindrical mounds with eight longitudinal streaks that are continuous with the tentacles. The mounds measure approximately 1.3–2.0 mm in diameter and 1.0–1.5 mm in height, with the diameter generally exceeding the height. Minute siphonozooids occur around the autozooids but are barely discernible to the naked eye (Fig. 1D).

***Axis***. The axis is solid, lacking internal cavities. The surface is smooth without longitudinal grooves. There are no pits at the bases of the polyps (Fig. 1C). The axis tapers toward the pointed branch tips, and a few commensal burrows are present along the axis (Fig. 1D).

***Colour***. According to the collector, the fresh coenenchyme was orange, with no colour difference from the autozooids. The dried coenenchyme and polyps are pale orange and the axis is pink; no colour variation was observed between the branch tips, base, or interior. Sclerites are a mixture of colourless and extremely pale orange.

**Figure 2.**
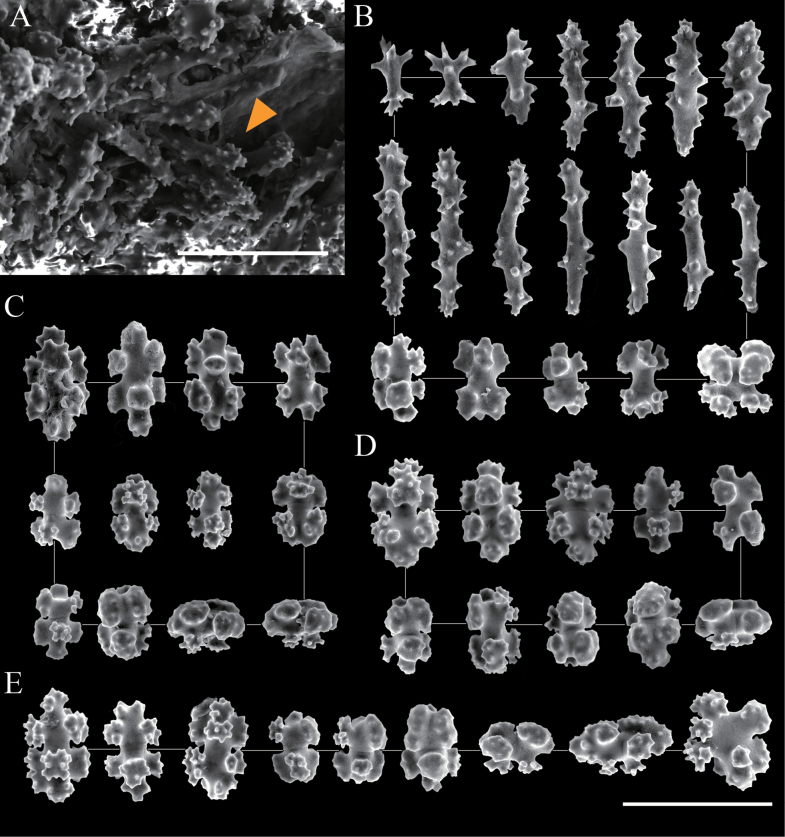
Scanning electron micrographs of sclerites of *Hemicorallium osmanthogemmum* sp. nov. (Holotype: KBF-OA-00436). **A**. Structure of autozooid; the arrow indicates rods from tentacle; **B**. Sclerites from tentacle; **C**. Sclerites from autozooid mound; **D**. Sclerites from coenenchyme at the branch tip; **E**. Sclerites from coenenchyme at the base of the colony. Scale bar: 100 μm.

**Figure 3.**
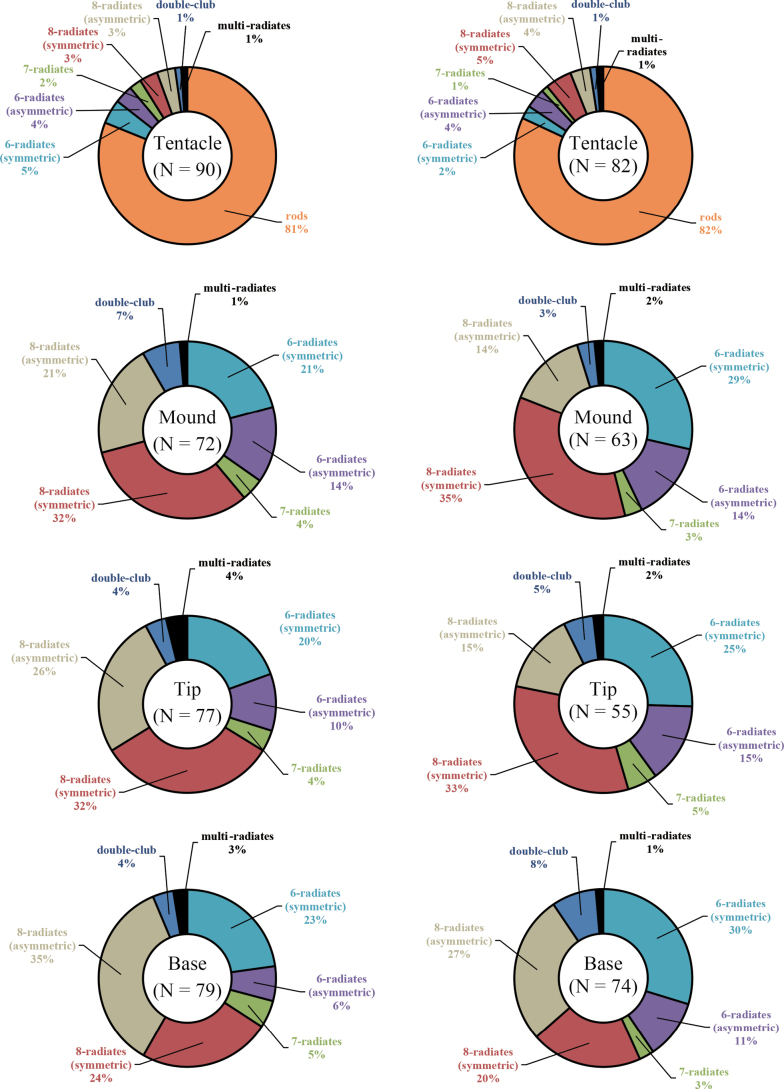
Composition of sclerites from each part sampled from *Hemicorallium osmanthogemmum* sp. nov., KBF-OA-00436 (left) and KBF-OA-00437 (right).

***Sclerites***. The sclerites comprise 6–8-radiates, double clubs, and rods. Coenenchymal sclerites are predominantly made up of 6- and 8-radiates, whereas rods occur exclusively on the tentacles where they predominate (Figs 2, 3). Double clubs and 7-radiates are rare throughout the colony. SEM measurements are as follows: rods are 0.060–0.124 mm long and 0.009–0.049 mm wide; 6-radiates are 0.051–0.056 mm × 0.034–0.037 mm; 7-radiates are 0.051–0.059 mm × 0.033–0.036 mm; 8-radiates are 0.057–0.087 mm × 0.031–0.041 mm; double clubs are 0.055–0.062 mm × 0.038–0.040 mm.

###### Variation.

**Figure 4.**
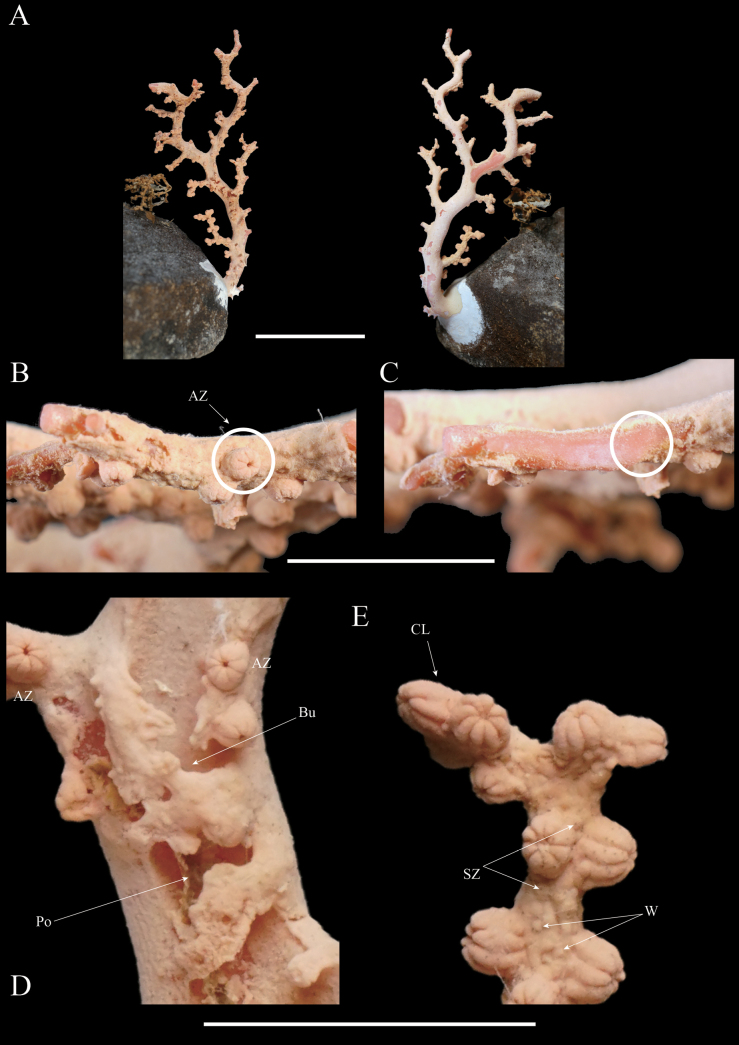
*Hemicorallium osmanthogemmum* sp. nov. (Paratype: KBF-OA-00437). **A**. ‘‘Front’’ and ‘‘back’’ of colony; **B**. Branch, showing autozooid (AZ); **C**. Exposed axis with the coenenchyme removed; **D, E**. Surface detail of branch, showing autozooid (AZ), siphonozooid (SZ), burrows (Bu), cluster of autozooids (CL), warts on coenenchyme surface (W), and commensal polychaete (Po). Scale bars: 50 mm (**A**); 10 mm (**B–E**).

**Figure 5.**
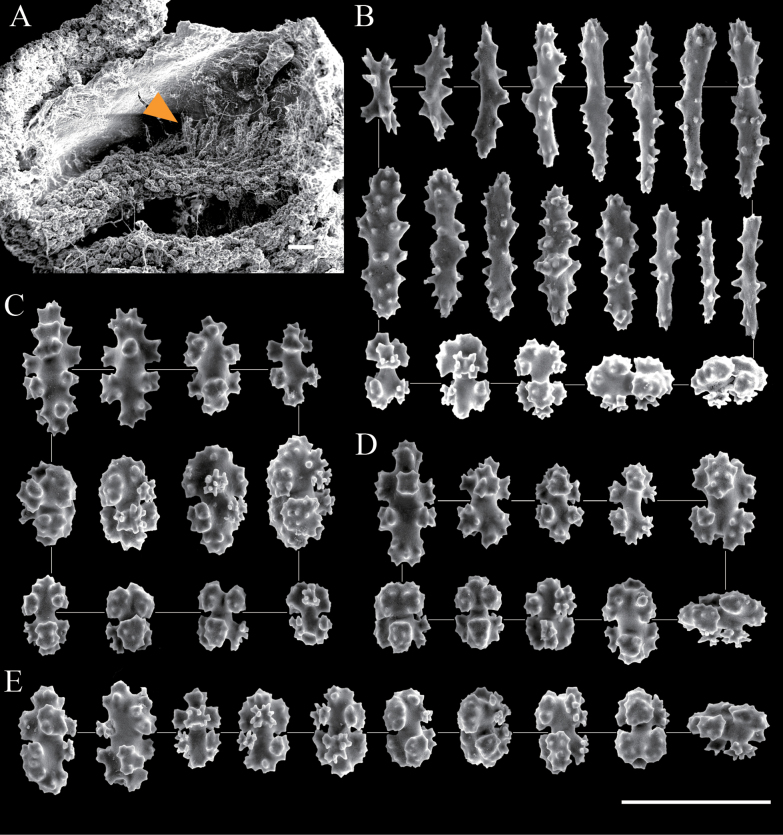
Scanning electron micrographs of sclerites of *Hemicorallium osmanthogemmum* sp. nov. (Paratype: KBF-OA-00437). **A**. Structure of autozooid; the arrow indicates rods from tentacle; **B**. Sclerites from tentacle; **C**. Sclerites from autozooid mound; **D**. Sclerites from coenenchyme at the branch tip; **E**. Sclerites from coenenchyme at the base of the colony. Scale bar: 100 μm.

Another specimen (KBF-OA-00437) was attached to a rock. Similar to the holotype, the colony branches in a single plane, with contracted autozooids distributed only on one side of the colony (Fig. 4A). Cluster formation occurs occasionally at the branch tips. Many terminal branches are broken and missing. The mounds are slightly larger than those of KBF-OA-00436, measuring 1.9–2.2 mm in diameter and 1.5–1.8 mm in height. The shape, maximum size, and dominant types of sclerites in each body part are consistent with those of KBF-OA-00436; however, the minimum sclerite size is smaller in KBF-OA-00437 (Fig. 5). The axis is slightly paler pink than that of specimen KBF-OA-00436, although it is still darker than the coenenchyme.

###### Remarks.

The 6- and 8-radiate sclerites can further be divided into “symmetric” and “asymmetric” forms. “Symmetric” forms have been considered an intermediate between radiates and double-club types (Nonaka and Hayashibara 2021; Nonaka et al. 2023; Hu et al. 2025); therefore, the boundaries between these three shapes are ambiguous. In this study, sclerites with two clearly defined handles were categorised as double-club types, following the criteria of Nonaka and Hayashibara (2021). Sclerites in the tentacles are predominantly rod shaped, whereas 6- and 8-radiate types dominate in the mounds, branch tips, and basal regions (Fig. 3). Among the previously described 24 species of *Hemicorallium*, *H. abyssale* (Bayer, 1956) and *H. sulcatum* (Kishinouye, 1903b) are morphologically similar to this species, particularly in possessing pink axial skeletons and double-club sclerites. However, neither of these species form clusters of autozooids at the branch tips, and in *H. abyssale*, autozooids are distributed on both sides of the colony, which contrasts with *H. osmanthogemmum* sp. nov. Seven species—*H. bathyrubrum* (Simpson & Watling, 2011); *H. ducale* (Bayer, 1955); *H. guttatum* Tu, Dai & Jeng, 2016; *H. indicodensum* Hu, Zhang & Xu, 2025; *H. jiaolongensis* Hu, Zhang & Xu, 2025; *H. meraboshi* Nonaka, Hanahara & Kakui, 2023; and *H. tricolor* (Johnson, 1899)—form autozooid clusters at the branch tips. However, none of these species have a pink axis, which differentiates *H. osmanthogemmum* sp. nov. from them. Additionally, the following six species cannot confidently be said to form autozooid clusters at branch tips based on the published descriptions: *H. aurantiacum* Tu, Dai & Jeng, 2016, *H. bayeri* (Simpson & Watling, 2011), *H. imperiale* (Bayer, 1955), *H. maderense* (Johnson, 1898), *H. niobe* (Bayer, 1964), and *H. variabile* (Thomson & Henderson, 1906). Among these, *H. bayeri*, *H. maderense*, *H. niobe*, and *H. variabile* can be distinguished from *H. osmanthogemmum* sp. nov. by their white axes, whereas *H. aurantiacum* and *H. imperiale* can be distinguished by their absence of double-club sclerites.

###### Etymology.

The specific name *osmanthogemmum* is derived from *Osmanthus* (sweet olive; Oleaceae) and the Latin *gemma*, meaning “bud.” This refers to the appearance of large autozooid mounds that are clustered at the distal ends of branches and resemble the floral buds of *Osmanthus*.

### Molecular phylogenetic results

**Figure 6.**
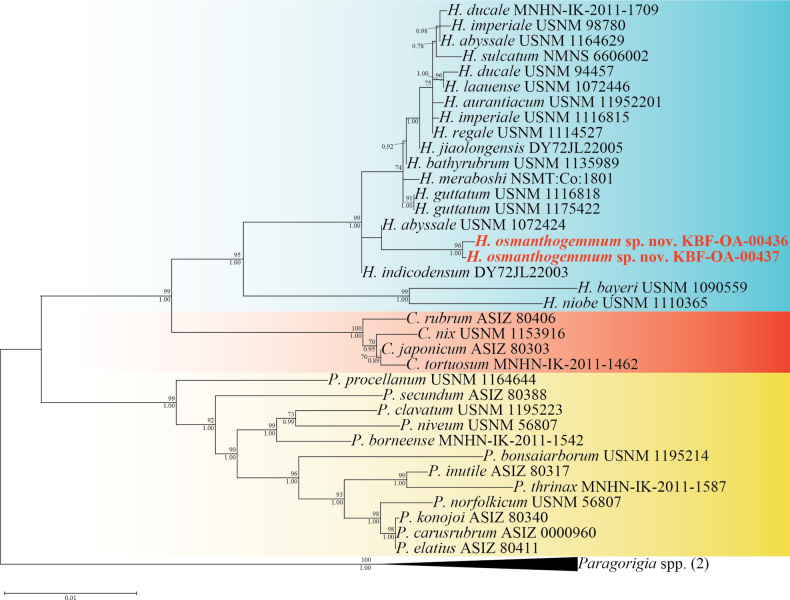
Phylogenetic relationships of species in the family Coralliidae based on the concatenated mitochondrial dataset (16S, ND1, ND2, ND3, ND6, COI, MSH, and IGR1). The tree was generated using Bayesian-inference and maximum-likelihood (ML) methods. ML bootstrap values (if > 70%) and Bayesian posterior probabilities (if > 90%) are shown at the nodes. The scale bar represents the number of substitutions per site. Each shaded colour indicates the genus: *Hemicorallium*, blue; *Corallium*, red; and *Pleurocorallium*, yellow.

A phylogenetic tree was constructed using ML and BI methods applied to a concatenated dataset of eight mitochondrial regions: 16S, ND1, ND2, ND3, ND6, COI, MSH, and IGR1 (Fig. 6). The molecular phylogenetic trees generated by ML and BI analyses showed similar topologies. Therefore, only the ML tree is shown here (Fig. 6). As in Tu et al. (2015b), Nonaka et al. (2023), and Hu et al. (2025), the genus *Hemicorallium* was recovered as a strongly supported monophyletic group using both ML and BI analyses (bootstrap value: 99%; posterior probability: 1). The two specimens analyzed in this study (KBF-OA-00436 and KBF-OA-00437) were clearly nested within the *Hemicorallium* clade (Fig. 6, blue shading). Furthermore, these specimens formed a lineage closely related to *H. abyssale* and *H. indicodensum*.

## Discussion

*Hemicorallium osmanthogemmum* sp. nov. (KBF-OA-00436 and KBF-OA-00437) exhibits diagnostic features of the genus *Hemicorallium*, which include nonretractile but contractile autozooids with an ovate-cylindrical shape and a sclerite assemblage comprising rods, crosses, 6-, 7-, and 8-radiates, and double clubs, with elongated rods or spindles found exclusively in the tentacles. However, this species is distinct from all previously described *Hemicorallium* species in several key features: the coenenchyme is pale orange, whereas the axis is pink; double-club sclerites are present; polyps are distributed only on one side of the colony; autozooid mounds are broader than they are tall; and the polyps tend to form clusters toward the distal ends of the branches. These trait combinations have not previously been reported in any known species of *Hemicorallium*. Furthermore, the results of molecular phylogenetic analyses using eight mitochondrial loci, including 16S, ND1, ND2, ND3, ND6, MSH, COI, and IGR1, showed that *H. osmanthogemmum* sp. nov. belongs to a clade that includes other members of the genus *Hemicorallium* and is clearly separated from all other species (Fig. 6). Therefore, based on both morphological and molecular phylogenetic analyses, this species is considered a new species of *Hemicorallium*.

Precious coral fisheries are operating in Kochi Prefecture; however, various restrictions have been imposed on collection methods to ensure resource conservation. One such regulation prohibits coral harvesting at depths greater than 200 m. Specimens of *H. osmanthogemmum* sp. nov. used in this study were collected as bycatch during a deep-sea *Beryx splendens* fishery off Cape Ashizuri at a depth of approximately 550 m. Although this species is unlikely to be targeted in future coral fisheries, its discovery highlights the presence of previously undocumented precious corals along the coast of Kochi Prefecture. These findings underscore the need for further investigations of the regional coral diversity.

### Key to the species of the genus *Hemicorallium*

This key includes 25 *Hemicorallium* species from the Pacific, Atlantic, and Indian Oceans. It is based on a revision of the keys provided by Tu et al. (2016), Nonaka and Hayashibara (2021), Hu et al. (2025), and Nonaka et al. (2025).

**Table:** 

1	Double clubs present	**2**
–	Double clubs absent	**13**
2	Coenenchyme yellow or white	**3**
–	Coenenchyme orange, pink, or reddish	**6**
3	Coenenchyme and autozooids of different colours	**4**
–	Coenenchyme and autozooids of the same colour	**5**
4	Coenenchyme creamy white; autozooids salmon-pink	***H. variabile* (Thomson & Henderson, 1906)**
–	Coenenchyme pale yellow; autozooids pale vermilion red	***H. tricolor* (Johnson, 1899)**
5	Autozooids densely distributed	***H. maderense* (Johnson, 1899)**
–	Autozooids sparsely distributed	***H. boshuense* (Kishinouye, 1903b)**
6	Autozooids biserially distributed	**7**
–	Autozooids distributed only (or mostly) on the front side	**8**
7	Autozooid mounds tall, up to 2.0 mm; asymmetric multi-radiates present	***H. abyssale* (Bayer, 1956)**
–	Autozooid mounds to 1.5 mm high and showing a considerable subdivision of the radiates or are otherwise misshapen	***H. ducale* (Bayer, 1955)**
8	Axis orange, pink, or red	**9**
–	Axis white	***H. bayeri* (Simpson & Watling, 2011)**
9	Autozooid mounds up to 2.0 mm high	**10**
–	Autozooid mounds approximately 2.5 mm high	***H. indicodensum* Hu, Zhang & Xu, 2025**
10	Autozooid mounds < 2.0 mm in diameter, no clustering	**11**
–	Autozooid mounds > 2.0 mm in diameter, clustered	***H. osmanthogemmum* sp. nov**.
11	Autozooid mounds similar in height and diameter; coenenchyme and axis orange to red	**12**
–	Autozooid mound height greater than diameter; coenenchyme and axis pink	***H. sulcatum* (Kishinouye, 1903b)**
12	Autozooid mounds small (0.9 mm in height, 1.15 mm in diameter) and dark pink	***H. taiwanicum* (Tu, Dai & Jeng, 2012)**
–	Autozooid mounds 1.4–1.5 mm in height and 1.3–1.5 mm in diameter; light orange	***H. reginae* (Hickson, 1905)**
13	Coenenchyme yellow or white	**14**
–	Coenenchyme orange, pink, or reddish when alive	**16**
14	Autozooid mound height greater than diameter; clustered	**15**
–	Autozooid mounds small and greater in diameter (1.5 mm) than in height (1.0 mm); no clustering	***H. laauense* (Bayer, 1956)**
15	Autozooids whitish or yellowish	***H. jiaolongensis* Hu, Zhang & Xu, 2025**
–	Autozooids pale vermilion red	***H. guttatum* Tu, Dai & Jeng, 2016**
16	Axis completely white	**17**
–	Axis orange, pink, or reddish	**18**
17	Coenenchyme pale red with a smooth surface	***H. meraboshi* Nonaka, Hanahara & Kakui, 2023**
–	Coenenchyme white to pink; small papillae often arranged in longitudinally oriented rows	***H. niobe* (Bayer, 1964)**
18	Sclerites are small, < 0.1 mm	**19**
–	Sclerites ≥ 0.1 mm in length	**21**
19	Coenenchyme surface without papillae	***H. aurantiacum* Tu, Dai & Jeng, 2016**
–	Coenenchyme surface granulated	**20**
20	Basal coenenchyme mainly contains 8-radiates (symmetric and asymmetric) > 0.05 mm long, with subordinate 6- and 7-radiates	***H. muzikae* Nonaka & Hayashibara, 2021**
–	Basal coenenchyme contains mainly 8-radiates (symmetric and asymmetric), 7-radiates, multi-radiates, and asymmetric 6-radiates, < 0.05 mm long	***H. tokiyasui* Nonaka & Hayashibara, 2021**
21	Autozooid mounds tall, up to 3.0 mm high	**22**
–	Autozooid mounds up to 2.0 mm high	**23**
22	Elongated 8-radiates reaching 0.1 mm in length present	***H. imperiale* (Bayer, 1955)**
–	Elongated 8-radiates not present (0.05–0.06 mm)	***H. halmaheirense* (Hickson, 1907)**
23	Coenenchyme pink or pale pink; autozooid mounds > 1.5 mm high	**24**
–	Coenenchyme red; autozooid mounds low (0.7–1.3 mm high)	***H. kaiyo* Nonaka & Hayashibara, 2021**
24	Axis deep pink to red	**25**
–	Axis pale pink; no clustering of autozooids	***H. regale* (Bayer, 1956)**
25	Autozooids clustered	***H. bathyrubrum* (Simpson & Watling, 2011)**
–	Autozooids without clustering	***H. ryukyuense* Nonaka, Takata & Yasuda, 2025**

## Supplementary Material

XML Treatment for Hemicorallium osmanthogemmum

## Additional information

### Artificial Intelligence (AI) use

The author accepts full responsibility for the content of the manuscript, including the disclosure of any use of AI.

No AI tools were used in the preparation of this manuscript.

### Author ORCIDs

T. Koido https://orcid.org/0000-0003-1004-5686

### Data availability

All of the data that support the findings of this study are available in the main text or Supplementary Information.

## Supplementary materials

Supplementary material 1 Summary of the genus *Hemicorallium* Data type xlsx Explanation note Summary of the genus *Hemicorallium* (Modified from Nonaka et al. 2023, 2025). Asterisks indicate species recorded outside the Western Pacific. Morphological characters inferred from the figures are underlined. https://binary.pensoft.net/file/1600417This dataset is made available under the Open Database License (http://opendatacommons.org/licenses/odbl/1.0/). The Open Database License (ODbL) is a license agreement intended to allow users to freely share, modify, and use this Dataset while maintaining this same freedom for others, provided that the original source and author(s) are credited. Tatsuki Koido
